# Alteration of serum bile acid profiles of HBV-related hepatocellular carcinoma identified by LC–MS/MS

**DOI:** 10.1007/s00432-024-05686-6

**Published:** 2024-03-25

**Authors:** Sijia Dai, Jingfei Zhu, Xuqiong Chen, Liming Zheng, Xiaoping Li, Longgen Liu

**Affiliations:** 1 Changzhou Clinical Medical College, Nanjing Medical University, 300 Lanling Road, Changzhou City, 213001 Jiangsu China; 2 Clinical Lab, Changzhou Third People’s Hospital, Changzhou Medical Center, Nanjing Medical University, Changzhou, 213001 China; 3 Institute of Hepatology, Changzhou Third People’s Hospital, Changzhou Medical Center, Nanjing Medical University, Changzhou, 213001 China

**Keywords:** Bile acids, Hepatitis B virus, Hepatocellular carcinoma, Liquid chromatography–mass spectrometry

## Abstract

**Background:**

Hepatocellular carcinoma closely related to metabolic disorders is a common and aggressive liver malignancy. The dysregulation of bile acid homeostasis has emerged as a key factor for the development and progression of HCC. We aimed to investigate the relationship between bile acids and HCC diagnosis and progression.

**Methods:**

A total of 744 HBV-related patients (including 396 HCC patients and 348 patients with chronic liver diseases) were enrolled in the current study. The baseline characteristics of patients were collected from electronic medical records, and the levels of bile acid profiles were determined by LC–MS/MS. Propensity score matching analysis was conducted to reduce the effect of selection bias, and receiver operating characteristic analysis was performed to evaluate the clinical application values of bile acid.

**Results:**

Significant differences were observed for most characteristics between the HCC group and the CLD group before PSM analysis. Patients with HCC were older and fatter (*p* < 0.05). After adjusting with a 1:1 ratio for age, gender and BMI, 42 HCC patients and 42 non-HCC patients were matched in 2 groups, respectively. The total bile acid level in HCC patients was lower than that in patients with chronic liver diseases before and after PSM analysis (*p* < 0.05). However, patients with HCC had significantly higher levels of DCA, LCA, and GLCA and lower levels of TCDCA, GUDCA, and TUDCA (*p* < 0.05, respectively). Besides, the TCDCA, TUDCA, GLCA, and GUDCA were significantly correlated with tumor procession. Moreover, the BAs profiles had a superior predictive ability for predicting the development of HCC even in patients with low serum AFP levels.

**Conclusion:**

Patients with HCC had significantly lower levels of total bile acid, but higher levels of secondary bile acids (DCA, LCA, and GLCA). The levels of primary bile acid (TCDCA) were closely related to tumor size and stage, which indicated that the bile acids were involved in the HCC procession and had important clinical application values.

**Supplementary Information:**

The online version contains supplementary material available at 10.1007/s00432-024-05686-6.

## Introduction

Hepatocellular carcinoma (HCC) is one of the most malignant tumors with the top third cancer-related death (8.2%) (Bray et al. 2018) worldwide. To date, the mortality rate of HCC is still rising globally (Wang et al. 2023). Various risk factors can lead to the development of HCC, such as hepatic viral infection, alcohol abuse, and metabolic dysfunction (Barcena-Varela and Lujambio 2021). The infection by hepatitis B and/or C virus remains the leading cause of liver cancer (about 40%) (Huang et al. 2022). Although frequently asymptomatic, the characteristics of metabolic perturbations prior to HCC may already be present and accompany the whole disease process (Yang et al. 2019). Accumulating evidence (Lamontagne et al. 2018) suggests that hepatitis virus infection may alter the host cell metabolism, involving glucose, lipid metabolism, and bile acid uptake.

Bile acids (BAs), which belong to cholesterol-derived sterols, were mainly synthesized in the liver and then secreted into the intestines to promote lipid absorption (Collins et al. 2023), as well as playing important roles in lipid and glucose metabolism and regulation of liver inflammation. The majority of conjugated bile acids are reabsorbed in the small intestine and returned to the liver, only a small fraction of primary bile acids are converted to secondary bile acids by gut bacteria (Thomas et al. 2022). Dysregulated or abnormally high levels of BAs are strongly associated with liver diseases and may alter the balance of gut microbiota (Xie et al. 2016). BAs are also involved in various cancer progression (Rezen et al. 2022). Chen et al*.* have found that bile acids could significantly promote the progression of HCC by activating the inflammasome (Chen et al. 2023). Moreover, considerably increased hepatic BAs were observed during the progression of high-fat diet-induced HCC (Xie et al. 2016). In addition, the profiles of BAs may be used as candidate metabolic biomarkers for the diagnosis of HCC (Xiao et al. 2012). Therefore, the vigorous changes of BAs may play an important role in promoting the development of HCC.

Although several studies have been conducted to explore the distribution of BAs in HCC, the deep BA profiles of early HBV-related HCC (diameter ≤ 2 cm and induced by HBV) have not been systematically studied or clarified to date. Hence, in this study, we examined the levels of serum BAs in patients with early HBV-related HCC to explore the bidirectional relationship using the liquid chromatography–mass spectrometry (LC–MS) method.

## Materials and methods

### Study population

Participants were derived from the patients hospitalized in the Third People’s Hospital of Changzhou between June 2022 and April 2023, including 396 HBV-related HCC and 348 non-HCC patients with chronic hepatitis B infection in this study. All patients enrolled in this study had a definite medical history of chronic HBV infection (HBsAg and HBV DNA positive, persistent or repeated elevation of ALT, and the course of the disease was more than 6 months) (Lok and McMahon 2009). Diagnostic criteria for all HCC patients were based on China Clinical Practice Guidelines (2019 Edition) (Department of Medical Administration and Health Commission of the People's Republic of 2020): (1) the nodules larger than 2 cm were diagnosed for HCC based on at least one typical HCC image (ultrasound, CT, MRI or angiography); (2) when the diameter of nodules was less than 2 cm, at least two imaging examinations have typical features of HCC; (3) the nodules were confirmed as HCC by histological examination. If any of the above items are met, HCC can be diagnosed. Exclusion criteria included (1) combined with other chronic liver diseases such as other hepatitis virus infections, alcoholic liver disease, autoimmune liver disease, and metabolic liver disease; (2) extrahepatic obstructive gall bladder diseases; (3) non-initial diagnosis of HCC or accompanied by other types of tumors; (4) incomplete baseline data.

### Laboratory methods

Serum samples were collected from HCC patients with initially diagnosed and stored at - 80 °C until analyzed. The routine liver function of patients was performed using an automatic biochemical instrument (HITACHI 7600, Japan), and the serum BAs profiles were measured by liquid chromatography with a tandem mass spectrometry system (LC–MS/MS; AB SCIEX 3200MD, USA). The brief detection procedure of BAs profiles is as follows: chromatographic separation was achieved on Waters BEH C18 column (1.7 µm, 100 × 2.1 mm internal dimensions) with gradient elution at a flow rate of 0.6 ml/min, using mobile phases of 0.05% acid ammonium formate in water (A) and acetonitrile (ACN) (B). The column temperature was maintained at 40 °C. The mass spectrometer was operated in the negative electrospray ionization mode. The voltage and temperature were set to -4500 V and 500 °C. The data acquisition was ascertained by Analyst MD 1.6.2 software. The accuracy was determined by replicate analysis of quality control samples (*n* = 6) at LQC (low-quality control) and HQC (high-quality control) levels. The % CV should be less than 15% and accuracy (% RE) should be within 15%. The isotopically labeled bile acids were used as an internal standard. The BAs profiles include primary bile acids and secondary bile acids, such as CA, CDCA, DCA, LCA, UDCA, TCA, TCDCA, TLCA, TDCA, TUDCA, GCA, GCDCA, GLCA, GDCA, GUDCA.

### Statistical analysis

All data were analyzed using SPSS software (version 26.0). Continuous variables were compared using Mann–Whitney *U* tests and were presented as median (interquartile range, IQR). Meanwhile, categorical variables were assessed using the Chi-square test and were described as the number of subjects (with percentages). The relationship between the relevant variables of the study was calculated using Spearman correlation coefficients. *p* value < 0.05 was considered as statistically significant.

## Results

### Characteristics of the study population

A total of 744 patients who met our selection criteria were enrolled in this study, including 396 HBV-related HCC patients and 348 non-HCC patients with chronic liver diseases (chronic HBV hepatitis or liver cirrhosis) (Table 1). Compared to the non-HCC patients, the HCC patients were older (59.44 versus 57) and fatter (23.85 versus 22.42); in addition, the liver function tests of HCC patients were better than those of patients with CLD. To reduce the effect of selection bias, we performed the propensity score matching (PSM) analysis with a 1:1 ratio using the nearest neighbor method for optimal balance of baseline characteristics, the variables used for matching were as follows: gender, age, and body mass index (BMI).

**Table 1:** The baseline characteristics of selected patients

Characteristics	Before PSM		*p value*	After PSM		*p value*
	HCC (*n* = 396)	CLD (*n* = 348)		HCC (*n* = 42)	CLD (*n* = 42)	
Age, ‾X ± SD, year	59.44 ± 10.52	57.00 ± 11.96	0.003	61.5 (55.5, 72.0)	63 (51.75, 72.25)	0.754
Gender, male, *n*, %	323 (81.6)	286 (82.2)	0.827	33 (78.6)	30 (71.4)	0.450
BMI, kg/m^2^	23.85 (21.27, 29.82)	22.42 (20.53, 25.78)	0.031	23.04 (21.37, 25.47)	22.82 (20.86, 24.56)	0.416
Cirrhosis	310 (78.3)	195 (56.0)	< 0.001	25 (59.5)	26 (61.9)	0.823
Liver function						
ALT, U/L	31.85 (21.68, 54.65)	44.30 (22.63, 135.00)	< 0.001	31.75 (19.85, 64.70)	35.80 (18.98, 97.15)	0.734
AST, U/L	34 (25, 64)	40 (25, 99.25)	0.010	38.5 (21.0, 91.25)	37.0 (24.75, 74.00)	0.626
ALP, U/L	103 (78, 147)	100.50 (76.25, 128.00)	0.028	102 (82.25, 166.50)	108.5 (62.5, 143.5)	0.403
GGT, U/L	61.30 (30.85, 153.80)	50.00 (27.00, 124.05)	0.024	58.45 (29.28, 189.40)	46.5 (28.28, 176.65)	0.677
Albumin, g/L	41.50 (35.83, 45.28)	39.85 (33.00, 44.40)	0.006	39.45 (32.88, 43.45)	32.20 (26.20, 39.93)	0.002
Total bilirubin, umol/L	17.0 (12.5, 26.5)	18.85 (13.20, 31.43)	0.022	17.20 (12.35, 27.95)	24.0 (12.98, 60.30)	0.149
Prothrombin time, s	14.1 (13.5, 15.0)	14.4 (13.4, 16.2)	0.012	13.95 (13.30, 14.85)	14.10 (13.10, 16.95)	0.408
Total bile acid, umol/L	8.9 (4.2, 23.2)	12.25 (4.6, 33.93)	< 0.001	7.35 (2.55, 28.05)	24.25 (7.25, 82.03)	0.028
Blood routine						
WBC, 10^9^/L	4.75 (3.56, 6.13)	4.81 (3.61, 5.98)	0.804	4.88 (3.68, 6.06)	4.46 (3.57, 5.97)	0.395
Neutrophil, 10^9^/L	2.83 (2.09, 3.79)	2.63 (1.94, 3.56)	0.047	2.91 (2.18, 3.94)	2.48 (1.97, 3.63)	0.243
Lymphocyte, 10^9^/L	1.18 (0.83, 1.69)	1.7 (0.88, 1.81)	0.007	1.14 (0.85, 1.49)	1.11 (0.62, 1.56)	0.635
RBC, 10^12^/L	4.41 (3.88, 4.86)	4.41 (3.80, 4.83)	0.418	4.30 (3.62, 4.58)	3.92 (3.04, 4.62)	0.177
Hemoglobin, g/L	139 (121, 151)	139 (118, 151)	0.268	131.5 (113.75, 144.25)	119 (103.25, 141.50)	0.100
Platelet, 10^9^/L	121 (84.25, 175.75)	123 (77.5, 187.0)	0.810	127 (95.50, 186.75)	121.5 (57.75, 200)	0.564
Tumor markers						
AFP, ng/ml	17.10 (3.93, 350.88)	3.85 (2.00, 17.08)	< 0.001	22.15 (2.50, 222.93)	2.50 (1.68, 4.15)	< 0.001
AFP-L3, %	10.05 (0.50, 43.53)	0.5 (0.5, 6.0)	< 0.001	12.35 (0.50, 44.58)	0.50 (0.50, 0.50)	< 0.001
PIVKAII, mAU/ml	116 (23, 1658.5)	15 (12, 20)	< 0.001	119 (23, 2131)	20.50 (15.75, 29.00)	< 0.001
Tumor stage, *n*, %						
Stage I	313 (79.0)			26 (61.9)		
Stage II	49 (12.4)			6 (14.3)		
Stage III	27 (6.8)			8 (19.0)		
Stage IV	7 (1.8)			2 (4.8)		
Tumor size, mm	32.40 (18.90, 54.73)			34.25 (17.83, 64.13)		

After PSM analysis, 42 HCC patients and 42 non-HCC patients were matched in 2 groups, respectively. The most general baseline characteristics of patients between the two groups were well balanced, but the serum levels of albumin and AFP were also significantly higher in the HCC group (*p* < 0.05, respectively). Interestingly, the total bile acid level in HCC patients was still lower than that in patients with chronic liver diseases (*p* < 0.05, respectively).

### The circulating bile acid profiles in matched patients

Since the levels of serum total BA were significantly different between the two groups before and after matching, we further explored the changes in circulating BA profiles. The LC–MS/MS results showed that the levels of DCA, LCA, and GLCA were significantly increased in patients with HBV-related HCC, and the levels of TCDCA, GUDCA, and TUDCA were lower (*p* < 0.05, respectively, Table 2).

**Table 2:** The circulating bile acid profilers in matched patients

	HCC (*n* = 42)	CLD (*n* = 42)	*p* value
Primary bile acids			
Cholic acid (CA), %	2.14 (0.51, 6.94)	1.29 (0.16, 4.84)	0.183
Glycocholic acid (GCA), %	14.59 (11.52, 19.54)	14.05 (8.01, 20.76)	0.502
Taurocholic acid (TCA), %	4.38 (2.00, 12.33)	4.61 (2.53, 11.05)	0.837
Chenodeoxycholic acid (CDCA),%	9.46 (0.99, 17.12)	6.05 (0.98, 16.69)	0.458
Glycochenodeoxycholic acid (GCDCA), %	46.30 (32.35, 56.79)	48.77 (35.23, 60.21)	0.352
Taurochenodeoxycholic acid (TCDCA), %	7.26 (4.07, 14.36)	14.41 (6.57, 18.10)	0.041
Secondary bile acids			
Deoxycholic acid (DCA), %	10.51 (0.00, 30.56)	0.08 (0.00, 6.05)	0.017
Glycodeoxycholic acid (GDCA), %	7.16 (0.00, 26.02)	0.00 (0.00, 24.08)	0.172
Taurodeoxycholic acid (TDCA), %	0.01 (0.00, 4.59)	0.01 (0.00, 3.17)	0.557
Lithocholic acid (LCA), %	1.02 (0.03, 2.68)	0.16 (0.00, 0.99)	0.020
Glycolithocholic acid (GLCA), %	1.54 (0.45, 2.55)	0.61 (0.09, 1.35)	0.008
Taurolithocholic acid (TLCA), %	0.31 (0.08, 0.72)	0.13 (0.05, 0.48)	0.126
Ursodeoxycholic acid (UDCA), %	9.00 (3.31, 25.80)	9.40 (3.66, 24.24)	0.886
Glycoursodeoxycholic acid (GUDCA), %	33.76 (14.31, 58.86)	65.34 (21.73, 72.49)	0.045
Tauroursodeoxycholic acid (TUDCA), %	1.54 (0.49, 7.46)	4.24 (1.15, 12.96)	0.043

### The correlation between bile acid profiles and tumor characteristics

**Fig. 1:**
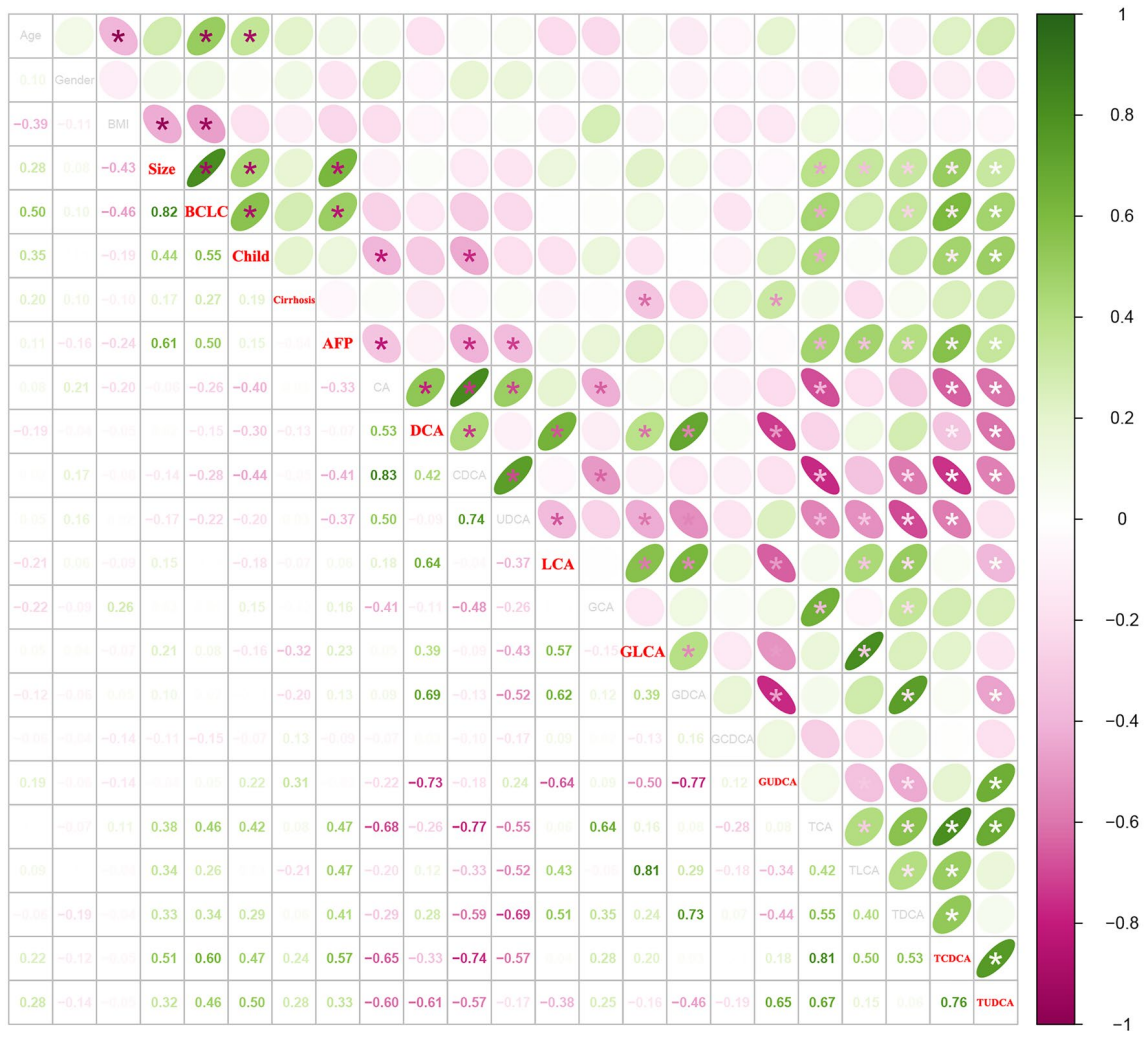
The correlation analysis between bile acid profiles and tumor characteristics. Colors indicate the degree of correlation, where green represents the positive correlation, red represents the negative correlation, the darker the color means the stronger the correlation. The “*” represents statistically significant results (*p* < 0.05), and the darker the color, the smaller the p value. The numbers in the left-bottom represent the Pearson’s correlation coefficient (*r*)

To gain further insights into the relationship between circulating BA profiles and tumor characteristics, we performed the correlation analyses using the Spearman method (Fig. 1). The levels of DCA had a negative association with the tumor stage (*r* =  − 0.15). Meanwhile, TCDCA and TUDCA showed a positive correlation with tumor size and tumor stage, respectively (*r* > 0.3, *p* < 0.05). Interestingly, the serum levels of GLCA and GUDCA also changed significantly in patients with cirrhosis (*p* < 0.05). In addition, the part of BA profiles demonstrated a strong association with the serum levels of AFP, such as CA, CDCA, UDCA, TCA, TDCA, TCDCA, and TUDCA (*r* > 0.3, *p* < 0.05, respectively). Therefore, the bile acid profiles may play an important role in the process of HCC.

### Clinical application value of bile acid profiles

**Fig. 2:**
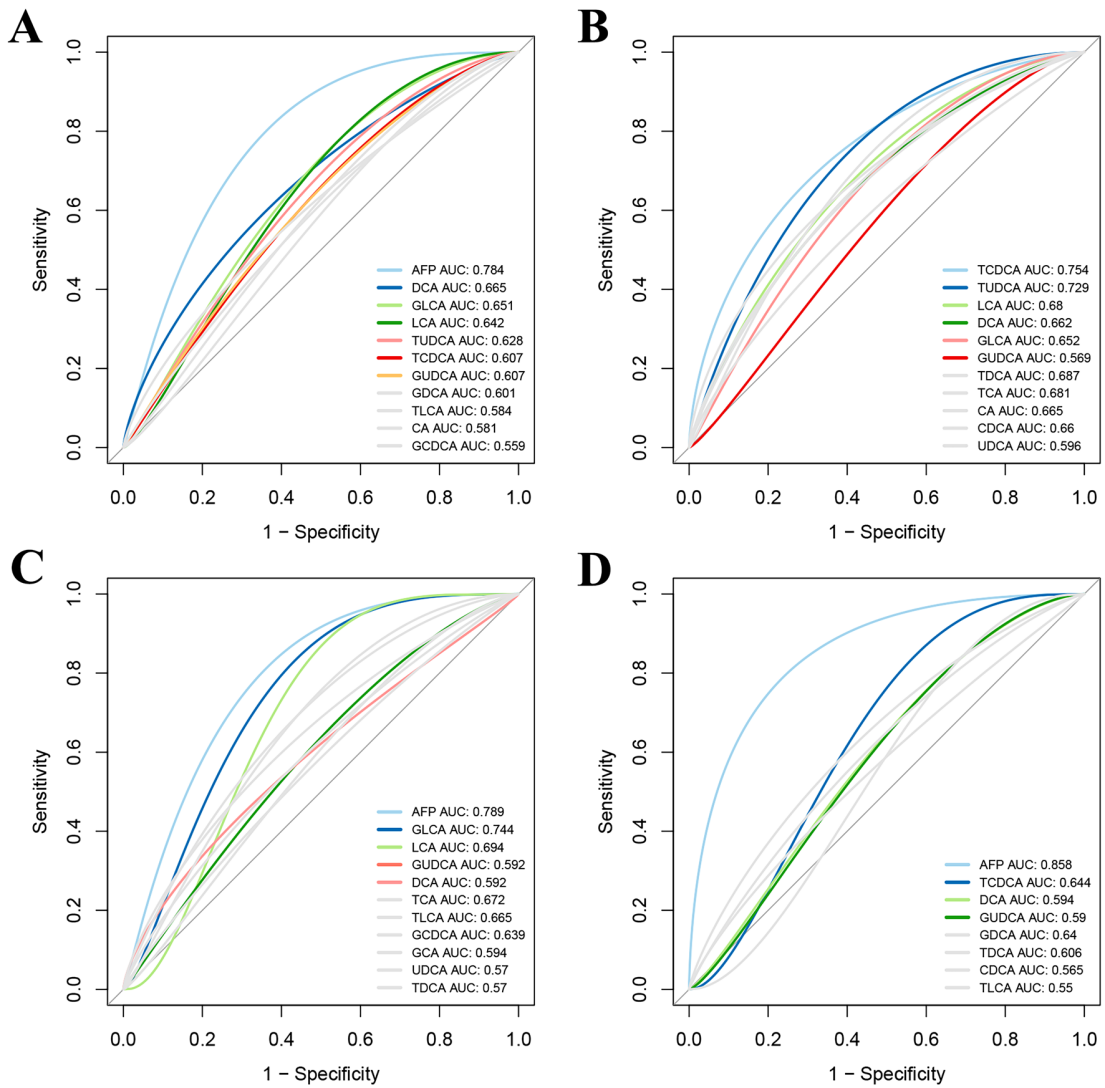
Clinical application value of bile acid profiles. **A** ROC curve analysis of bile acid profiles for predicting the HCC development. **B** ROC curve analysis of bile acid profiles for predicting the HCC development in patients with low AFP levels. **C** ROC curve analysis of bile acid profiles for predicting the HCC development in patients with Child A stage. **D** ROC curve analysis of bile acid profiles for predicting the HCC development in patients with Child B/C stage

Since the BA profiles demonstrated a strong association with the tumor process, we further analyzed the clinical application values using receiver operating characteristic (ROC) curve analyses. The AUC values demonstrated that the BAs profiles had a superior predictive ability for predicting the development of HCC even in patients with low serum AFP levels (AFP <  = 20 ng/ml; AUC value > 0.55, respectively), although it was weaker compared with AFP (Fig. 2A, and B). Notably, the primary bile acids TCDCA and the secondary bile acids DCA/GLCA have higher predictive values, suggesting the changes in the BA profiles were associated with the development of HCC. Furthermore, the BAs profiles still had an excellent ability to independently predict values regardless of the liver functional reserve (Fig. 2C, and D). In conclusion, the BA profiles play an important role in the progression of HCC and have important clinical application values.

## Discussion

Long-term stimulation of chronic HBV infection can play a significant role in tumor-promoting effects through varietal mechanisms (Jiang et al. 2021). Metabolic reprogramming is increasingly recognized as a key contributing to the development of HCC (Luo et al. 2022). The deregulation of glucose, fatty acid, amino acid, and glutamine metabolism is critically involved in the development and progression of cancer (Du et al. 2022). As the final products of cholesterol catabolism, bile acids are essential for the absorption, transport, and metabolism of lipids (Li and Chiang 2014). Furthermore, bile acids have a diverse array of functions to regulate cellular metabolic, inflammatory, and proliferative phenotypes (Colosimo and Tomlinson 2022). An important determinant of the biological effects of bile acids is their ability to activate a variety of receptors and mediate downstream signals. Such as FXR and TGR5 are the two most important receptors for BA mediation (Luo et al. 2022). FXR is expressed mainly in hepatocytes, while TGR5 is expressed primarily in Kupffer cells and liver sinusoidal cells (Wang et al. 2013). Some studies have reported that FXR activity could inhibit the occurrence of HCC, suppressing BA reuptake to hepatocytes and hepatic inflammation, and directly increasing the expression of tumor‑suppressor genes (Huang et al. 2015; Ananthanarayanan et al. 2001). TGR5 can also suppress the progression of hepatic inflammation and cancer (Wang et al. 2013). Changes in bile acid homeostasis and bile acid-mediated signaling pathways may affect hepatic metabolic homeostasis and lead to hepatocarcinogenesis, while CDCA is the most potent FXR agonist and LCA is the most potent TGR5 ligand (Rezen et al. 2022).

In the current study, we performed the PSM analysis to reduce the effect of selection bias and explored the circulating bile acid profiles using LC–MS techniques. The results showed that patients with HCC had significantly lower levels of total bile acid, while patients with HCC had significantly higher levels of secondary bile acids, such as DCA, LCA, and GLCA. Moreover, the distribution of TCDCA, TUDCA, GLCA, and GUDCA was significantly correlated with tumor precession. In addition, bile acid profiles showed superior predictive ability for HCC development even in patients with low serum AFP levels, suggesting that these bile acid profiles may play an important role in the development of HCC, which may be used as indicators to evaluate the progression of liver cancer. Taken together, the results of our study add to the growing body of evidence for further revealing the development of HCC.

The relationship between bile acids and HCC has garnered increasing interest in recent years due to its not only playing an important role in lipid metabolism but also in maintaining the homeostasis of gut microbiota. Examining bile acids profiles in previous studies of HCC have been reported, although the results were not entirely consistent. Tan Y et al. (2012), using a non-target metabolomics method to detect the serum metabolic profiling, showed that TCA was an effective biomarker for the discrimination of small liver tumors (tumor size less than 2 cm) with 80.5% sensitivity and 80.1% specificity, and also enabling the differentiation HCC from patients with chronic liver disease. However, the serum level of TCA was not abnormal in the current study. Petrick JL et al. (2020) found that higher concentrations of conjugated primary bile acids were associated with an increased risk of HBV- and HCV-related HCC. A study has shown that TCDCA can significantly increase the proliferation of hepatoma cells and decrease the expression of CEBP α (a tumor suppressor protein) (Xie et al. 2016). Similarly, excessive LCA or DCA was closely positively associated with the development of HCC (Amaral et al. 2009), which was also consistent with the findings of the current study. However, the inconsistent findings in the literature could be related to different geographic regions, limited sample sizes, and statistical adjustment for covariates.

Certain mechanisms may be involved in the relationship between bile acids and the progression of HBV-related HCC. The NLRP3 inflammasome is a critical component of the innate immune system (Kelley et al. 2019). Yu et al. demonstrated that HBV inhibits NLRP3 inflammasome activation by suppressing the NF-κB pathway and ROS production, which contributes to HBV-induced immunotolerance (Yu et al. 2017). Immune tolerance could help the virus evade immune surveillance and promote the progression of the disease from inflammation to tumor formation (Jiang et al. 2021). Different bile acids have different regulatory effects on the activity of the NLRP3 inflammatory body (Guan et al. 2022), which suggests that the change of bile acid profiles may affect the activity of NLRP3 inflammatory bodies, and then affect the progress of HBV-related HCC.

The study’s main strengths included no significant differences in age, sex, or body mass index between the two groups, and all bile acids were calculated as a percentage to reduce the effect of individual differences. We studied the relationship between bile acids and the size or stage of the tumor and found some valuable biomarkers, which can provide some help for the clinical stage diagnosis of HCC. However, this study still has some inevitable limitations. First, the cross-sectional study design is difficult to reflect the dynamic progress of individual diseases. Second, due to the limitations of clinical research, we did not take into account the genetic risk factors, virus-related risk factors, and the medical history of patients. Third, we only studied the association between HBV-induced HCC and CLD in the absence of healthy controls, and the number of populations included in this study was insufficient.

In conclusion, we characterized the metabolic profiles of 15 bile acids in serum levels of patients with HBV-induced HCC and CLD. DCA, LCA, and GLCA were found to be significantly elevated in HCC patients. TCDCA and TUDCA were correlated with the tumor size, which may be helpful to the diagnosis and staging of HCC. However, the clinical efficacy of these biomarkers still needs to be confirmed by large-scale studies, and the potential mechanisms need to be explored through precise experiments.

### Supplementary Information

Below is the link to the electronic supplementary material.

Supplementary file1 (XLSX 53 KB)

## Data Availability

The datasets generated during and/or analyzed during the current study are available from the corresponding author on reasonable request.

## Abbreviations

HCC: Hepatocellular carcinoma
CLD: Chronic liver diseases
HBV: Hepatitis B virus
BA: Bile acid
LC–MS/MS: Liquid chromatography with a tandem mass spectrometry system
PSM: Propensity score matching
CA: Cholic acid
GCA: Glycocholic acid
TCA: Taurocholic acid
CDCA: Chenodeoxycholic acid
GCDCA: Glycochenodeoxycholic acid
TCDCA: Taurochenodeoxycholic acid
DCA: Deoxycholic acid
GDCA: Glycodeoxycholic acid
TDCA: Taurodeoxycholic acid
LCA: Lithocholic acid
GLCA: Glycolithocholic acid
TLCA: Taurolithocholic acid
UDCA: Ursodeoxycholic acid
GUDCA: Glycoursodeoxycholic acid
TUDCA: Tauroursodeoxycholic acid
FXR: Farnesoid X receptor
TGR5: Takeda G protein-coupled receptor 5

## References

[CR1] Amaral JD, Viana RJ, Ramalho RM (2009). Bile acids: regulation of apoptosis by ursodeoxycholic acid. J Lipid Res.

[CR2] Ananthanarayanan M, Balasubramanian N, Makishima M (2001). Human bile salt export pump promoter is transactivated by the farnesoid X receptor/bile acid receptor. J Biol Chem.

[CR3] Barcena-Varela MA (2021). The endless sources of hepatocellular carcinoma heterogeneity. Cancers (basel).

[CR4] Bray F, Ferlay J, Soerjomataram I (2018). Global cancer statistics 2018: GLOBOCAN estimates of incidence and mortality worldwide for 36 cancers in 185 countries. CA Cancer J Clin.

[CR5] Chen W, Ding M, Ji L (2023). Bile acids promote the development of HCC by activating inflammasome. Hepatol Commun.

[CR6] Collins SL, Stine JG, Bisanz JE (2023). Bile acids and the gut microbiota: metabolic interactions and impacts on disease. Nat Rev Microbiol.

[CR7] Colosimo SandTomlinson JW (2022). Bile acids as drivers and biomarkers of hepatocellular carcinoma. World J Hepatol.

[CR8] Department of Medical Administration N HandHealth Commission of the People's Republic of C (2020). Guidelines for diagnosis and treatment of primary liver cancer in China (2019 edition). Zhonghua Gan Zang Bing Za Zhi.

[CR9] Du D, Liu C, Qin M (2022). Metabolic dysregulation and emerging therapeutical targets for hepatocellular carcinoma. Acta Pharm Sin B.

[CR10] Guan B, Tong J, Hao H (2022). Bile acid coordinates microbiota homeostasis and systemic immunometabolism in cardiometabolic diseases. Acta Pharmaceutica Sinica B.

[CR11] Huang X-f, W-d Z-y (2015). FXR and liver carcinogenesis. Acta Pharmacol Sin.

[CR12] Huang DQ, Singal AG, Kono Y (2022). Changing global epidemiology of liver cancer from 2010 to 2019: NASH is the fastest growing cause of liver cancer. Cell Metab.

[CR13] Jiang Y, Han Q, Zhao H (2021). The mechanisms of HBV-induced hepatocellular carcinoma. J Hepatocell Carcinoma.

[CR14] Kelley N, Jeltema D, Duan Y (2019). The NLRP3 inflammasome: an overview of mechanisms of activation and regulation. Int J Mol Sci.

[CR15] Lamontagne RJ, CandBouchard CJ, M J.  (2018). A broad investigation of the HBV-mediated changes to primary hepatocyte physiology reveals HBV significantly alters metabolic pathways. Metabolism.

[CR16] Li TandChiang JYL (2014). Bile acid signaling in metabolic disease and drug therapy. Pharmacol Rev.

[CR17] Lok ASF, McMahon BJ (2009). Chronic hepatitis B: update 2009. Hepatology.

[CR18] Luo W, Guo S, Zhou Y (2022). Hepatocellular carcinoma: novel understandings and therapeutic strategies based on bile acids (Review). Int J Oncol.

[CR19] Petrick JL, Florio AA, Koshiol J (2020). Prediagnostic concentrations of circulating bile acids and hepatocellular carcinoma risk: REVEAL-HBV and HCV studies. Int J Cancer.

[CR20] Rezen T, Rozman D, Kovacs T (2022). The role of bile acids in carcinogenesis. Cell Mol Life Sci.

[CR22] Tan Y, Yin P, Tang L (2012). Metabolomics study of stepwise hepatocarcinogenesis from the model rats to patients: potential biomarkers effective for small hepatocellular carcinoma diagnosis. Mol Cell Proteom.

[CR23] Thomas JP, Modos D, Rushbrook SM (2022). The emerging role of bile acids in the pathogenesis of inflammatory bowel disease. Front Immunol.

[CR24] Wang X, Fu X, Van Ness C (2013). Bile acid receptors and liver cancer. Curr Pathobiol Rep.

[CR25] Wang J, Xu H, Wang Y (2023). Efficacy and safety of drug-eluting bead TACE in the treatment of primary or secondary liver cancer. Can J Gastroenterol Hepatol.

[CR26] Xiao JF, Varghese RS, Zhou B (2012). LC-MS based serum metabolomics for identification of hepatocellular carcinoma biomarkers in Egyptian cohort. J Proteome Res.

[CR27] Xie G, Wang X, Huang F (2016). Dysregulated hepatic bile acids collaboratively promote liver carcinogenesis. Int J Cancer.

[CR28] Yang YM, Kim S, YandSeki E (2019). Inflammation and liver cancer: molecular mechanisms and therapeutic targets. Semin Liver Dis.

[CR29] Yu X, Lan P, Hou X (2017). HBV inhibits LPS-induced NLRP3 inflammasome activation and IL-1β production via suppressing the NF-κB pathway and ROS production. J Hepatol.

